# Evaluation of the revised versus the simplified scoring system in patients with autoimmune hepatitis

**DOI:** 10.3892/etm.2013.1366

**Published:** 2013-10-25

**Authors:** YI LI, MILIN PENG, GUOZHONG GONG

**Affiliations:** Institute of Hepatology, Second Xiangya Hospital, Central South University, Changsha, Hunan 410011, P.R. China

**Keywords:** autoimmune hepatitis, Chinese, diagnosis system, evaluation

## Abstract

The aim of this study was to evaluate the simplified and revised scoring systems for the diagnosis of autoimmune hepatitis (AIH). Seventy-seven patients diagnosed with AIH via the revised scoring system were enrolled in this study. Statistical analysis was performed by means of the χ^2^ test and logistic regression analysis. A total of 39 patients with definite AIH and 38 patients with probable AIH were diagnosed by the revised scoring system, whereas among these 77 patients, the simplified scoring system classified nine cases as definite AIH, 39 as probable AIH and 29 without AIH. In this study, the parameters contributing to the discrepant diagnosis of AIH were compared using the revised and simplified systems. A χ^2^ test showed that antinuclear antibody (ANA) or smooth muscle antibody (SMA) titers were significantly lower in the patients with discrepant diagnoses (χ^2^=15.0, P=0.001). Logistic regression with backward selection revealed that for the discrepant diagnosis of patients, the presence of other concurrent autoimmune diseases [odds ratio (OR)=7.25; P=0.018; 95% confidence interval (CI), 1.41–37.29] was the only independent risk factor. In addition, the presence of anti-soluble liver antigen/liver-pancreas antigen (SLA/LP) or perinuclear antineutrophil cytoplasmic antibody (pANCA) (OR=0.12; P=0.022; 95% CI, 0.02–0.74), the level of immunoglobulin G (IgG) with 1–1.1 × Normal (N) (OR=0.02; P=0.044; 95% CI, 0.00–0.89) and ANA or SMA titers ≥1:80 (OR=0.04; P=<0.001; 95% CI, 0.01–0.23) were three independent protective factors. In conclusion, the revised scoring system has a superior performance in the diagnosis of patients with AIH compared with the simplified scoring system. According to the simplified scoring system, other concurrent autoimmune diseases are the risk factor for the AIH diagnosis.

## Introduction

Autoimmune hepatitis (AIH) was first described in 1950 as a progressive liver disease of unknown cause (1). The disease is characterized by interface hepatitis on histological examination, hyper immunoglobulin G (IgG) and autoantibodies (2–4). The absence of specific clinical presentation and serological markers in AIH patients may make a correct and timely diagnosis difficult (5). The International Autoimmune Hepatitis Group (IAIHG) proposed the diagnostic criteria for AIH and a diagnostic scoring system in 1993 (6), which were subsequently revised in 1999 (7). The merit of the revised scoring system is that it is capable of diagnosing individuals who lack certain classical features (hypergammaglobulinemia or autoantibodies) or who exhibit atypical manifestations (antimitochondrial antibodies, cholestasis or atypical histological features). However, the diagnostic criteria have been criticized since they are complex (13 components and 29 possible grades) and are not widely available as an easily applicable clinical tool (7–9). As a result, a simplified scoring system based on four components [level of autoantibody expression, serum IgG concentration, liver histology and the absence of viral markers] was developed in 2008 by the IAIHG (10). In a retrospective study, the simplified scoring system performed with a sensitivity of 95% and a specificity of 90% (11). Czaja (11) analyzed 153 individuals with AIH by codified clinical criteria and concluded that the performance parameters of each scoring system were the same. The selected patients from previous studies were including not only AIH patients but also primary biliary cirrhosis (PBC) or primary sclerosing cholangitis (PSC) patients, and, to the best of our knowledge, no recent study has evaluated the independent factors that affect the diagnostic discrepancy between the revised and simplified scoring systems, particularly in Asia. The aim of this study was to evaluate the independent parameters associated with the diagnostic discrepancy between the two scoring systems by analyzing the clinical and laboratory characteristics of 77 patients with AIH.

## Patients and methods

A retrospective analysis was performed of the patients diagnosed with definite or probable AIH, according to the revised criteria of the IAIHG in 1999, in the Second Xiangya Hospital (Changsha, China) over a nine-year period (2002–2011). For each patient, age, gender, clinical presentation, the prevalence of concurrent autoimmune diseases, laboratory and immunological data, and serological markers of viral hepatitis were obtained. The patients were enrolled in the present study prior to undergoing specific therapy. Liver biopsy results were also obtained. Patients with viral liver diseases, hereditary hemochromatosis, Wilson’s disease, nonalcoholic fatty liver disease (NFLD), PBC and PSC were excluded. Thia study was approved by the Ethics Committee of Central South University (Changsha, China). The procedures were in accordance with the ethical standards of the Committee on Human Experimentation of Central South University and/or were performed in accordance with the Helsinki Declaration of 1975. Informed consent was obtained from all patients.

Serum IgG concentration was evaluated using immunonephelometry. In addition, hepatitis B serum markers (HBsAg, HBsAb, HBcAb, HBeAb and HBeAg), Hepatitis A Virus IgM antibody (HAVAb-IgM) and antibodies to hepatitis C and E were assessed in the patients using second-generation enzyme-linked immunosorbent assays (ELISAs). Smooth muscle antibody (SMA), antineutrophil cytoplasmic antibody (ANCA) and antinuclear antibody (ANA) levels were examined using indirect immunofluorescence (IIF). Antibodies to liver kidney microsome type 1 (LKM-1), antimitochondrial antibodies (AMA) and soluble liver antigen/liver-pancreas antigen (SLA/LP) were assessed using ELISA.

Liver tissue examinations were performed in 33 patients, and the liver specimens were evaluated by two pathologists who specifically identified the characteristic histological features defined by the IAIHG, including interface hepatitis, lymphoplasmacytic infiltrate, liver cell rosettes and biliary changes. Professor H.P. Dienes (Institute of Pathology, University of Cologne, Cologne, Germany) and Professor A.W. Lohse (Department of Medicine, Johannes Gutenberg-University, Mainz, Germany) defined three categories for grading histology: Atypical histology, histology compatible with AIH and typical histology. Interface hepatitis, lymphocytic/lymphoplasmacytic infiltrates in portal tracts and extending into the lobule, emperipolesis (active penetration by one cell into and through a larger cell) and hepatic rosette formation were regarded as typical for the diagnosis of AIH. To be considered typical, each of the three features of typical AIH histology had to be present. Compatible features consisted of chronic hepatitis with lymphocytic infiltration without all the features considered typical. Histology was considered atypical when showing signs of another diagnosis, such as steatohepatitis.

The revised scoring system (as the ‘gold standard’) and the simplified scoring system were used to provide diagnoses of definite, probable or negative for AIH in the patients. According to the revised criteria, a pretreatment score of 10–15 points indicated probable AIH and >15 points indicated definite AIH. In the simplified scoring system, a score ≥6 and <7 points supported probable AIH and a score ≥7 points supported a diagnosis of definite AIH.

### Statistical analysis

Continuous data are expressed as a percentage and mean ± standard deviation (SD). The χ^2^ test or Fisher’s exact test were used for the comparison of single parameters. Logistic regression models were utilized to evaluate factors associated with discrepancies in the AIH diagnosis between the revised and the simplified criteria. A backward elimination strategy was used to identify factors independently associated with disease; the probability for removal was set at 0.15. Variables included in the analysis were gender, age, concurrent autoimmune disease, serum IgG concentration, alkaline phosphatase (ALP)/aspartate aminotransferase (AST) levels, AMA, the presence and level of autoantibody expression [ANA or SMA and SLA/LP or perinuclear antineutrophil cytoplasmic antibody (pANCA)], liver histology (compatible or typical), viral markers and drug history. Data were analyzed using the SPSS statistical software package version 17.0 (SPSS, Chicago, IL, USA). During statistical analysis, serum ALP:AST ratios were categorized into <1.5, 1.5–3 and >3, and serum IgG levels were transformed into Normal (N) group, 1–1.1 × N group and >1.1 × N group, and the titers of ANA or SMA were grouped into <1:40, 1:80>x≥1:40 and ≥1:80. The results are presented as odds ratios (ORs), 95% confidence intervals (CIs), Wald values and P-values. P≤0.05 was considered to indicate a statistically significant difference.

## Results

### Clinical profile and laboratory findings in patients with AIH

A total of 77 patients, including 67 females (87%) and 10 males (13%), who were classified as having probable or definite AIH via the IAIHG revised scoring system were identified as those who also had complete clinical, laboratory and histological data. Patient age ranged between 10 and 78 years (mean ± SD, 50.4±14.7). Other associated autoimmune diseases were observed in 44 patients (57.1%). Among the 44 patients, systemic lupus erythematosus was the most common disorder [18 patients (23.4%)]. Autoimmune thyroiditis was present in five patients (6.5%), Sjögren’s syndrome in nine (11.7%), diabetes in seven (9.1%), ulcerative colitis in one (1.3%) and rheumatoid arthritis in three (3.9%).

With regard to biochemical data, conventional liver function tests, serum IgG concentration and coherent antibodies were assessed prior to the treatment of the patients. Sixty-two of the 77 patients (80.5%) showed high serum IgG levels of >2,500 mg/dl. Fifty-nine patients (76.6%) had an ALP:AST ratio of <1.5. Fifty patients (70.1%) were positive for ANA, including 29 patients (37.7%) with titers of ≥1:80. Anti-SLA/LP tests were positive in 13 patients (16.9%) and pANCA was positive in nine patients (11.7%).

Histological biopsies were conducted prior to treatment, and histological assessments were performed for 33 patients (42.9%). The results indicated that 33 patients had interface hepatitis. Plasma cell infiltration in the portal area was observed in 18 patients and three had liver cell rosettes. According to the simplified scoring system, compatible histological manifestation was observed in 30 patients and typical in three patients.

### Comparison of the two scoring systems for the diagnosis of AIH

**Figure 1:**
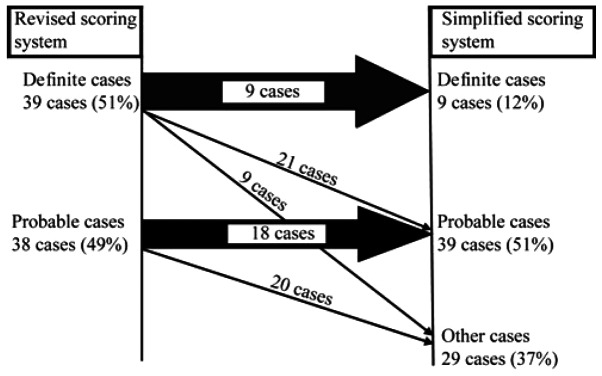
Discrepant autoimmune hepatitis diagnosis based on the revised and simplified criteria.

The revised scoring system was applied to 77 patients prior to treatment. The revised system graded 39 subjects (50.6%) with the diagnosis of definite AIH and 38 subjects (49.4%) with probable AIH. By contrast, the simplified scoring system diagnosed 48 patients (62.3%) with AIH. Of the 39 patients with definite AIH diagnosed by the revised criteria, the simplified criteria classified nine as definite AIH, 21 as probable AIH and nine without AIH. Of the 38 patients with probable AIH diagnosed by the revised scoring system, 20 patients were classified as being without AIH by the simplified scoring system (Fig. 1). The concordance rate between the two scoring systems was 62.3% (48/77). Among the 77 patients, the results indicated that 50 patients (64.9%) had a discrepant AIH diagnosis and 27 patients had an accordant diagnosis by the two scoring systems. A χ^2^ test was applied to evaluate the clinical characteristics of the 77 patients and compare the single parameter that affected the diagnosis when using the revised scoring system and the simplified scoring system. With regard to the revised scoring system, patients with definite AIH had higher titers of ANA or SMA (χ^2^=10.08, P=0.01) than those diagnosed with probable AIH. In addition, whether the patients underwent a liver biopsy (χ^2^=5.93, P=0.021) was also a statistically significant factor affecting the diagnosis. Using the simplified scoring system, patients with a definite or probable diagnosis had higher levels of IgG (χ^2^=17.35, P=0.002) and higher titers of ANA or SMA (χ^2^=34.63, P<0.001) than those diagnosed without AIH. The clinical and laboratory features of the 77 patients diagnosed using the revised scoring system and the simplified scoring system are listed in Tables I and II.

### Factors influencing the diagnostic discrepancy in the scoring systems

The characteristics of the 50 patients with discrepant diagnoses were as follows: Six (12.0%) were male, 31 (62.0%) had other concurrent autoimmune diseases, 39 (78.0%) had ALP/AST ratios of <1.5, 24 (48.0%) had low ANA titers (<1:40), 28 (56.0%) did not undergo liver biopsy and 10 (20%) had normal levels of IgG.

The patients were divided into two groups (accordant and discrepant diagnoses using the revised and simplified scoring systems) to compare those parameters that contributed to the discordant diagnoses of the two scoring systems.

According to the statistical analysis, single factor analysis (χ^2^ test or Fisher’s exact test) showed that patients with a discrepant diagnosis had lower titers of ANA or SMA (<1:40) than those with accordant diagnosis (48 vs. 18.5%, χ^2^=15.0, P=0.001). In addition, patients with a discordant diagnosis presented more frequently with compatible histology than those with an accordant diagnosis (44 vs. 29.6%, χ^2^=6.5, P=0.038). The data are listed in Table III.

The parameters were subsequently entered into the logistic regression model to determine which independent factor was associated with diagnostic discordance. The results are presented in Table IV. The analysis showed that the presence of concurrent autoimmune diseases (OR=7.25; P=0.018; 95% CI, 1.41–37.29) was the only important independent risk factor associated with the discrepant diagnosis by the scoring systems. However, the presence of anti-SLA/LP or pANCA (OR=0.12; P=0.022; 95% CI, 0.02–0.74), the level of IgG with 1–1.1 × N (OR=0.02; P=0.044; 95% CI, 0.00–0.89) and titers of ANA or SMA of ≥1:80 (OR=0.04; P<0.001; 95% CI, 0.01–0.23) were three independent protective factors. However, the presence of normal or >1.1 × N levels of IgG, titers of ANA or SMA of ≥1:40, liver histology and whether the patient underwent a liver biopsy were not statistically significant factors contributing to discrepant diagnoses when using the revised and simplified scoring systems (P>0.05).

## Discussion

Previous studies have demonstrated that compared with the revised scoring system, the simplified scoring system exhibits high sensitivity and specificity for the diagnosis of definite or probable AIH, and even superior specificity and predictability for the disease (11–14). The study by Qiu *et al*(15), supported the simplified criteria for the diagnosis of patients, noting that the simplified criteria had high sensitivity and specificity for the diagnosis of AIH. The study also showed that lower IgG levels and less frequent positivity for autoantibodies or lower titers may be two causes contributing to downgraded or excluded diagnosis by the simplified criteria. Although the sensitivity and specificity was predicted, the parameters affecting the discordant diagnosis by the simplified criteria have not, until now, been independently evaluated in patients.

Czaja (11) suggested that the simplified scoring system may underestimate the diagnosis of AIH in patients who have few or atypical features of the disease. In the present patient cohort, the application of the simplified criteria was less likely to ascribe a definite diagnosis of AIH than the revised scoring system. The concordance rate between the two scoring systems was 62.3%. Fifty of the 77 patients (64.9%) identified as having AIH by the revised scoring system were downgraded by the simplified scoring system. Twenty-four of the 50 patients with a discrepant diagnosis who were downgraded by the simplified scoring system had low ANA titers (<1:40). Patients with accordant diagnoses had higher titers of ANA than those with discrepant diagnosis by the two scoring systems. From the logistic regression analysis, titers of ANA of ≥1:80 and the presence of anti-SLA/LP or pANCA were likely to decrease the ratio of downgraded risk via the simplified scoring system. In addition, IgG levels of 1–1.1 × N was an independent protective factors; however, these levels were not considered to be statistically significant with regard to discordant diagnosis. Therefore, it was suggested that lower or higher IgG levels were not the main determinants that affected the discrepancies in diagnosis. This differed from the study by Qiu *et al*(15), in which low IgG levels were one of the main discriminants that downgraded or even excluded from the diagnosis of AIH by the simplified scoring system.

The present study attempted to identify certain notable clinical features in the patients with AIH. Notably, another discrepancy between the scoring systems was the presence of other concurrent autoimmune diseases (excluding liver diseases). Of the AIH patients diagnosed by the revised scoring system, 57.1% had other associated autoimmune diseases. This presence ratio was higher than that observed in other regions. García-Torres *et al*(16) revealed that associated autoimmune diseases were observed in 24.7%of patients with AIH in Spain. According to a nationwide survey in Japan (17), the prevalence of complicating Sjögren syndrome in patients with AIH was ~10%. Furthermore, 62% patients with a discordant diagnosis by the two scoring systems had other concurrent autoimmune diseases. The present study further showed that the presence of other concurrent autoimmune diseases increases the risk of discrepant diagnosis by the two scoring systems. The revised scoring system may facilitate the diagnosis of AIH, rather than that of other autoimmune diseases, in cases that are unrecognized by the simplified scoring system. In China, the detection of other associated autoimmune diseases is a pivotal factor in the diagnosis of AIH. A previous study (14) showed that the simplified scoring system excluded the diagnosis of other diseases with concurrent immune features more frequently than the revised scoring system.

Certain studies (13,15) demonstrated that liver histological scores were critical for making the differential diagnosis between AIH and other chronic liver diseases. However, in the present study, it was revealed that liver biopsy was not a factor contributing to the discrepant diagnosis by the two scoring systems. In this study, liver histology with compatible or typical manifestation may enhance the diagnosis with AIH via the simplified scoring system.

In the present study, the revised scoring system performed better in the patients than the simplified criteria. The revised scoring system was developed to support the diagnosis of AIH in patients who lacked the conventional serological markers of the disease and particularly in the patients with other associated autoimmune diseases. Its golden standard in this regard remains unchallenged (18). Therefore, the revised scoring system was capable of diagnosing AIH in those patients who were downgraded or unrecognized using the simplified scoring system. Despite this, the simplified scoring system was utilized as a convenient tool for the four parameters, and was capable of easily classifying the diagnosis. Notably in the present study, which was performed in China, the patients with AIH had various characteristics that differed from patients from other regions, particularly Europe (19,20). Those features indicate that the simplified scoring system may be less sensitive than the revised scoring system in ascribing an overall (probable or definite) diagnosis of AIH (62.3 vs. 100%). However, the two scoring systems were created to support the clinical diagnosis, not to supersede each other. The combination of systems may ensure a correct diagnosis of patients with AIH.

In conclusion, in this study it was concluded that other concurrent autoimmune diseases present frequently in Chinese patients with AIH. The presence of other concurrent autoimmune diseases, the high titer of ANA or SMA and the presence of other autoantibodies are important in the discrepant diagnosis of AIH by the two scoring systems. The golden standard of the revised scoring system remains unchallenged. Due to the convenience of the simplified scoring system, it was suggested that this system may be utilized as a subordinate tool, and that the revised scoring system should be applied to enhance the diagnosis of AIH.

## Figures and Tables

**Table I:** Comparison of the clinical features of 77 patients with definite and probable AIH diagnoses according to the revised scoring system.

Parameters	Definite AIH (score >15) (n=39)	Probable AIH (score 10–15) (n=38)	χ^2^	P-value
Female	34	33	0.002	0.965
Concurrent autoimmune diseases	23	21	0.108	0.820
ALP:AST ratio			0.004	0.998
1.5–3	5	5		
<1.5	30	29		
IgG			4.293	0.117
Normal	3	8		
Abnormal	36	30		
ANA or SMA			10.085	0.010
≥1:80>x≥1:40	13	6		
≥1:80	18	11		
Anti-SLA/LP or pANCA	7	7	0.003	1.000
Liver histology	22	11	5.927	0.021

AIH, autoimmune hepatitis; ALP, alkaline phosphatase; AST, aspartate aminotransferase; IgG, immunoglobulin G; ANA, antinuclear antibody; SLA/LP, soluble liver antigen/liver-pancreas antigen; pANCA, perinuclear antineutrophil cytoplasmic antibody.

**Table II:** Comparison of the clinical features of 77 patients with diagnoses of definite, probable and no AIH according to the simplified scoring system.

Parameters	Definite (score ≥7) (n=9)	Probable (score 6≤x<7) (n=39)	Other (score <6) (n=29)	χ^2^	P-value
Female	8	33	26	0.406	0.816
Concurrent autoimmune diseases	5	16	12	0.672	0.715
ALP:AST ratio				3.881	0.422
1.5–3	0	7	3		
<1.5	7	28	24		
IgG				17.348	0.002
Normal	1	0	10		
Abnormal	8	39	19		
ANA or SMA				34.627	<0.001
1:80>x≥1:40	2	2	2		
≥1:80	7	37	17		
Anti-SLA/LP or pANCA	2	9	3	1.924	0.382

AIH, autoimmune hepatitis; ALP, alkaline phosphatase; AST, aspartate aminotransferase; IgG, immunoglobulin G; ANA, antinuclear antibody; SLA/LP, soluble liver antigen/liver-pancreas antigen; pANCA, perinuclear antineutrophil cytoplasmic antibody.

**Table III:** Comparison of important parameters between 77 patients with discrepant and accordant diagnoses by χ^2^ test.

Parameters	Discrepant diagnoses (n=50)	Accordant diagnoses (n=27)	χ^2^	P-value
Female	44	23	0.123	0.734
Concurrent autoimmune diseases	31	13	1.374	0.335
ALP:AST ratio			0.932	0.628
1.5–3	7	3		
<1.5	39	20		
IgG			4.013	0.134
Normal	10	1		
Abnormal	40	26		
ANA or SMA			15.000	0.001
1:80>x≥1:40	15	4		
≥1:80	11	18		
Anti-SLA/LP or pANCA	8	6	0.456	0.499
Liver histology			6.517	0.038
Not tested	28	16		
Compatible	22	8		
Typical	0	3		

ALP, alkaline phosphatase; AST, aspartate aminotransferase; IgG, immunoglobulin G; ANA, antinuclear antibody; SLA/LP, soluble liver antigen/liver-pancreas antigen; pANCA, perinuclear antineutrophil cytoplasmic antibody.

**Table IV:** Logistic regression analysis of independent factors associating with a discrepant diagnosis by the two systems.

Parameters	B	Odds ratios (OR)	Wald value	P-value	95% CI
Drug history	24.68	5.24E10	0.00	1.000	0.00
Concurrent autoimmune diseases	1.98	7.25	5.62	0.018	1.41–37.29
IgG					
Normal	4.12	0.128			
1–1.1 × Normal	−3.96	0.02	4.07	0.044	0.00–0.89
>1.1 × Normal	−2.05	0.13	2.38	0.123	0.01–1.73
ANA or SMA					
<1:40		14.40	0.001		
1:80>x≥1:40	0.41	1.51	0.18	0.676	0.22–10.26
≥1:80	−3.32	0.04	12.27	<0.001	0.01–0.23
Anti-SLA/LP or pANCA	−2.15	0.12	5.24	0.022	0.02–0.74
Liver histology					
Not tested	0.59	0.743			
Compatible	−0.68	0.51	0.59	0.441	0.09–2.84
Typical	−21.95	0.00	0.00	0.999	0.00

B, regression coefficient; CI, confidence interval; IgG, immunoglobulin G; ANA, antinuclear antibody; SMA, smooth muscle antibody; SLA/LP, soluble liver antigen/liver-pancreas antigen; pANCA, perinuclear antineutrophil cytoplasmic antibody.
